# Catecholaminergic Gene Variants: Contribution in ADHD and Associated Comorbid Attributes in the Eastern Indian Probands

**DOI:** 10.1155/2013/918410

**Published:** 2013-09-19

**Authors:** Paramita Ghosh, Kanyakumarika Sarkar, Nipa Bhaduri, Anirban Ray, Keka Sarkar, Swagata Sinha, Kanchan Mukhopadhyay

**Affiliations:** ^1^Manovikas Biomedical Research and Diagnostic Centre, 482 Madudah, Plot I-24, Sector-J, E.M. Bypass, Kolkata 700107, India; ^2^Department of Biotechnology, CT Institute of Pharmaceutical Sciences, Jalandhar, Panjab 140020, India; ^3^Chembiotek, TCG Lifesciences, Kolkata 700091, India; ^4^Department of Psychiatry, Chittaranjan National Medical College, Kolkata 700020, India

## Abstract

Contribution of genes in attention deficit hyperactivity disorder (ADHD) has been explored in various populations, and several genes were speculated to contribute small but additive effects. We have assessed variants in four genes, DDC (rs3837091 and rs3735273), DRD2 (rs1800496, rs1801028, and rs1799732), DRD4 (rs4646984 and rs4646983), and COMT (rs165599 and rs740603) in Indian ADHD subjects with comorbid attributes. Cases were recruited following the Diagnostic and Statistical Manual for Mental Disorders-IV-TR after obtaining informed written consent. DNA isolated from peripheral blood leukocytes of ADHD probands (*N* = 170), their parents (*N* = 310), and ethnically matched controls (*n* = 180) was used for genotyping followed by population- and family-based analyses by the UNPHASED program. DRD4 sites showed significant difference in allelic frequencies by case-control analysis, while DDC and COMT exhibited bias in familial transmission (*P* < 0.05). rs3837091 “AGAG,” rs3735273 “A,” rs1799732 “C,” rs740603 “G,” rs165599 “G” and single repeat alleles of rs4646984/rs4646983 showed positive correlation with co-morbid characteristics (*P* < 0.05). Multi dimensionality reduction analysis of case-control data revealed significant interactive effects of all four genes (*P* < 0.001), while family-based data showed interaction between DDC and DRD2 (*P* = 0.04). This first study on these gene variants in Indo-Caucasoid ADHD probands and associated co-morbid conditions indicates altered dopaminergic neurotransmission in ADHD.

## 1. Introduction

Attention deficit hyperactivity disorder (ADHD) is a neurodevelopmental disorder characterized by age inappropriate inattentiveness, hyperactivity, and impulsivity [1]. Comorbidity is quite common in ADHD with around 60–100% patients exhibiting one or more co-morbid conditions. Among different co-morbid characteristics, oppositional defiant disorder (ODD), conduct disorder (CD), anxiety disorder (AD), depressive disorder, mood disorder (MD), and learning disabilities (LD) are of frequent occurrence [2]; around 27% of ADHD patients were reported to have ODD and/or CD, while 18% had AD. The Diagnostic and Statistical Manual for Mental Disorders-IV-text revised (DSM-IV-TR) describes ADHD children with ODD as unusually disobedient and hostile towards higher authority [1]. A number of children with ADHD (46%) were also reported to have LD, experiencing difficulty in reading, spelling, vocabulary, arithmetic, and written communication [2]; this affects not only academics but also their social lives. Common frontal lobe dysfunction was observed in both ADHD and LD patients [3]. 

Strong genetic basis of ADHD is supported by twin, adoption, or family-based studies [4, 5]. A major role of genes regulating neurotransmitters, leading mainly to dopamine (DA) dysfunction, has been postulated in the disease etiology (reviewed in [6]). Since DA activity is essential to the motor and cognitive functioning of the brain, a wide range of neurological symptoms were speculated from malfunctioning of even a single part of the system [7]. 

Action of DA is mediated through DA receptors (DRD) grouped in two families based on the activation (D1-like receptors, DRD1 and DRD5) or inhibition (D2-like receptors, DRD2, DRD3, and DRD4) of adenylate cyclase in response to ligand binding [8, 9]. The dopaminergic hypothesis of ADHD is based mostly on the malfunctioning of D2-like receptors in the brain [10, 11]. The DRD4 gene encoding for DA receptor 4 has been extensively studied, and positive associations with ADHD were reported in Caucasian as well as several non-Caucasian populations [5, 12–15]. The D2 and D3 receptors have been studied mostly in Caucasian and Chinese populations revealing inconsistent findings [16–18].

Genes encoding for enzymes involved in the catecholaminergic system like catechol-O-methyl transferase (COMT) [19, 20], dopamine decarboxylase (DDC) [21–23], dopamine beta hydroxylase [5, 15], and monoamine oxidase [15, 18, 22, 23] have also gained a lot of importance in exploring the etiological basis of ADHD. Some of these genetic variants have revealed significant association with ADHD associated co-morbid disorders [24] and were speculated as the reason for comorbidities being so common in Caucasian subjects with ADHD [25]. 

In the Indo-Caucasoid ADHD probands, DRD4, DAT1, MAOA, COMT, and DBH gene variants showed significant association with the disorder [5]. However, till date, neither any report on DDC and DRD2 nor any information on the contribution of gene variants in ADHD associated co-morbid disorders was available in this particular ethnic group. Since ADHD subjects frequently exhibit co-morbid behavioral disorders including CD, ODD, and substance abuse [2, 3], in the present study we have selected nine polymorphic sites in four catecholaminergic genes, DRD2, DRD4, DDC, and COMT, which modulate function of DA. The sites analyzed have been explored in association with ADHD in European Caucasian and Han Chinese populations [12–14, 18, 22, 25–28] or behavioral disorders [29, 30] and analyzed for the first time for their contribution in the etiology of Indo-Caucasoid ADHD probands stratified on the basis of different co-morbid disorders. 

## 2. Subjects and Methods

### 2.1. Study Subjects

We recruited ADHD probands (*N* = 170) from the out-patient department of Manovikas Kendra Rehabilitation and Research Institute for the Handicapped, Kolkata, based on (a) DSM-IV-TR criteria [1]; (b) hyperactivity level measured by Conners' Parents and Teachers Rating Scale [31]; (c) intelligence/developmental quotient assessed by Wechsler's Intelligence Scale for Children [32] for children above five years and Developmental Screening Test for children below 5 years [33]. Mean age of probands was 7.7 years ± 2.3 SD, and male to female ratio was 10 : 1. Out of 170 probands, 143 were complete parent-proband trios, 17 had only one parent, and 10 were affected probands only. Hyperactive/impulsive (11.2%) and inattentive (7.3%) subtypes were only few, while majority of the probands belonged to the combined subtype (81.5%). Based on the presenting co-morbid symptoms, ADHD probands were subgrouped as ADHD-comorbidity, ADHD + CD, ADHD + LD, ADHD + ODD, and ADHD + MD. Subjects suffering from only psychiatric problems, pervasive developmental disorders, and any form of mental retardation (IQ ≤ 80) including fragile X syndrome were excluded. 

A control group (*N* = 180; mean age 19.7 years ± 7.94 SD; and male to female ratio 10 : 3), evaluated following the DSM-IV-TR criteria [1] for ADHD, was also recruited. All the individuals enlisted for the study belonged to the Indo-Caucasoid ethnic category. For participation in the study, informed written consent was obtained from the controls and guardians of ADHD probands. The study protocol was approved by the Institutional Human Ethical Committee. 

### 2.2. Selection of SNPs and Genotyping

Nine polymorphic sites in four genes, that is, DDC (rs3837091 (AGAG Ins→Del) and rs3735273 (G→A)), DRD2 (rs1800496 (C→T), rs1801028 (C→G), and rs1799732 (C→Del)), DRD4 (rs4646984 (429 bp/549 bp) and rs4646983 (286 bp/298 bp)), and COMT (rs740603 (G→A) and rs165599 (G→A)), were selected based on their association with ADHD [12–14, 18, 22, 25–28] or behavioral disorders [29, 30] in other ethnic groups. Functional role of these sites was obtained from published literature. Sites without any published report were analyzed by F-SNP (http://compbio.cs.queensu.ca/F-SNP/).

Peripheral blood was collected from ADHD probands, their parents and controls for isolation of genomic DNA [34]. Details of oligonucleotide sequences and amplification protocols are provided in Table S1, in Supplementary Material available online at http://dx.doi.org/10.1155/2013/918410.

### 2.3. Statistical Analysis

Data obtained was subjected to both population-as well as family-based association analyses. The GENEPOP program (web version 3.4) (http://wbiomed.curtin.edu.au/genepop/) was used to calculate allelic and genotypic frequencies followed by analyses for Hardy-Weinberg equilibrium (HWE). For case-control analysis, we have used the program COCAPHASE, which is a part of a suite of programs UNPHASED [35]; allele/genotype frequencies of each marker obtained for the control individuals were compared with these of the ADHD case group and their parents. For analysis of family-based transmission, Extended-transmission disequilibrium test (ETDT) [36] which is also a part of UNPHASED, was used. In this program, transmission from a single heterozygous (informative) parent (duos) to an affected individual can be used for calculation. Different groups with co-morbid characteristics were analyzed separately to find out association with the comorbidity. Since numbers of cases were small after stratification based on co-morbid characteristics, for this analysis, we have used the haplotype-based haplotype relative risk (HHRR) program under the UNPHASED; transmission from informative as well as noninformative parents is taken into account for HHRR [37]. Comparisons were tested for multiple corrections (1000 itinerations) while running the UNPHASED. Data showing significant association were further checked for power of the test by Piface version 1.72 [38]. Odds ratio calculator was used to calculate the odds ratio (OR) and its confidence interval (www.hutchon.net/ConfidOR.htm). Relative risk calculator was used to calculate the relative risk (RR) and its confidence interval (http://www.hutchon.net/ConfidRR.htm). While OR portrays the strength of association between two binary data values compared symmetrically, RR describes the likelihood of developing disease in an exposed group compared to a nonexposed group. 

### 2.4. Epistatic Interaction

Multifactor dimensionality reduction (MDR) program [39] was used for analysis of the case-control data set. Tuned ReliefF filter algorithm [40] was used to screen noisy polymorphisms. Since the number of affected and unaffected individuals was not equal in the present dataset, balanced accuracy with random seed 1 was used to avoid spurious results due to chance divisions of the data [41]. Then a naive Bayes classifier in the context of a 10-fold cross validation was used to estimate the testing accuracy of each one dimensional attribute of the 2-factor to 10-factor models. The cross-validation consistency (CVC) was also calculated, which measures the number of times, out of 10 divisions of the data, when the same best model was found [42]. The model with the maximum testing balanced accuracy (TBA), a CVC > 5 out of 10, and a minimum prediction error (PE)/misclassification error for that comparison was considered as the best model [42]. Statistical significance (*P* values) was calculated using a 1000-fold permutation test to compare observed testing accuracies with those expected under the null hypothesis of no association. 

For the family-based data, we have analyzed only the trio families by MDR phenomics version 1.0 [43]. In absence of any phenotype, the MDR-pedigree disequilibrium test (MDR-PDT) was used for analysis [44]; the missing genotype was coded as “3” in the input file. Statistical significance was calculated after a 1000-fold permutation test. *P* values for each statistic were obtained by fixed (FixP, does not control for multiple tests) and nonfixed permutation tests (Non-FixP, controlling for multiple testing).

## 3. Results

rs1800496 and rs1801028 were found to be nonpolymorphic after analyzing 100 control subjects and 30 families with ADHD probands; only the “C” variant was detected (Table 1), and we did not perform any further analysis for these sites. Control genotypes for rs165599 deviated marginally from the equilibrium (Table 2), while other sites studied obeyed the HWE in all the groups (Table 2).

Case-control analysis exhibited significantly higher frequency of the single repeat allele of rs4646983 in ADHD cases (Table 1). Parents of ADHD probands showed higher allelic frequencies for both rs4646983 and rs4646984 (Table 1). rs4646983 showed only a trend for higher significance (*P* = 0.09), which could be due to absence of homozygous genotype of the single repeat variant in the control subjects (Table 2). Other sites failed to show any significant difference in allelic (Table 1) as well as genotypic frequencies (Table 2).

Family-based TDT analysis (Table 3) revealed significant bias in transmission of rs3837091 “AGAG” (*P* = 0.01, power ~75% alpha at 5%). Further analysis revealed that this bias was due to maternal overtransmission of the “AGAG” allele, more specifically to male probands (Table S2, *P* = 0.01, and power ~85% alpha at 5%). rs740603 “G” (*P* = 0.02, power ~65% alpha at 5%) also showed a bias in transmission to ADHD cases (Table 3); this bias was due to paternal overtransmission (Table S2). 

Haplotype analysis showed lower frequency of rs3837091-3735273 “Del-G” in ADHD cases (Table S3) which could be primarily due to significant nontransmission (*P* = 0.001, power ~90% alpha at 5%) of this haplotype from the parents (Table S4). The rs4646983-rs4646984 2R-2R haplotype was present predominantly in control individuals (Table S3). Haplotype “G-A” of rs165599-rs740603 exhibited higher transmission (*P* = 0.04, power ~57% alpha at 5%) to ADHD probands (Table S4). 

Major comorbidities observed in ADHD children from eastern India are LD (44%), ODD (33%), CD (31%), and MD (16%). Substance abuse disorder, tic disorder, and AD were found in only few cases and excluded from further analysis. Comparative analysis of ADHD probands subgrouped on the basis of co-morbid characteristics revealed the following observations.

### 3.1. *DDC*

By population-based analysis, we have noticed significant differences in “AGAG” allele frequency for rs3837091 in ADHD comorbidity (Table 4); the “AGAG/AGAG” genotype was also overrepresented in this group (*χ* ^2^ = 7.6; *P* = 0.02). Further, there was an overtransmission of the “AGAG” allele (*P* = 0.006) (Table 5), which was principally paternal in nature (*χ* ^2^ = 5.78, *P* = 0.02, power ~23% alpha at 5%). On the other hand, maternal overtransmission of the “AGAG” allele was significant in ADHD + CD (*χ* ^2^ = 5.3, *P* = 0.02, and power ~22% alpha at 5%). In ADHD + MD, “AGAG/AGAG” genotype showed lower frequencies in parents as well as probands as compared to the control population (*χ* ^2^ = 26.4 and 6.3; *P* < 0.0001 and 0.04 for probands and parents, resp.).

rs3735273 showed significant differences in allelic and genotypic frequencies in ADHD + CD in comparison to controls; “A” allele and “AA” genotype frequencies were higher in probands (*χ* ^2^ = 6.5 and 12.3; *P* = 0.01 and 0.002 resp.) OR was also high in this group (2.17).

Family-based analysis failed to show any significant bias in transmission for rs3735273 (Table 5). 

### 3.2. *DRD2*

Population-based analysis (Table 4) revealed significant differences in allelic and genotypic frequencies for rs1799732; the “C” allele (*χ* ^2^ = 4.64; *P* = 0.03) and “CC” genotype were overrepresented in ADHD + LD (*χ* ^2^ = 9.68; *P* = 0.008) as well as in ADHD + MD (genotypic *χ* ^2^ = 5.86; *P* = 0.05) with a noticeably high OR. Family-based analyses showed overtransmission of the “C” allele (Table 5) to ADHD + LD (*χ* ^2^ = 7.49, *P* = 0.006; OR = 6.33, power = 79%  *α* at 5%). Other comorbidities failed to show any significant contribution (Tables 4 and 5).

### 3.3. *DRD4*

In ADHD + CD, the “single repeat” (1R) allele of rs4646984 showed higher frequencies (*P* = 0.04) as compared to the control population (Table 4) along with significant (*P* = 0.02) familial overtransmission (Table 5).

rs4646983 also showed significant differences in allelic (1R) and genotypic (1R1R) frequencies in ADHD + CD (*χ* ^2^ = 4.70 and 7.78; *P* = 0.03 and 0.02, resp.), ADHD + ODD (*χ* ^2^ = 4.75, 13.6; *P* = 0.03, 0.001, resp.), and ADHD + MD (*χ* ^2^ = 4.29 and 20.9; *P* = 0.04 and 0.001, resp.) by population-based analysis (Table 4); the OR was above 2 in all the co-morbid groups. Family-based analysis showed lack of any transmission bias (Table 5); though OR was high in ADHD + CD and ADHD + MD, it could be due to a wide variation in confidence interval.

### 3.4. *COMT*

The rs165599 “G” allele was found to be significantly overrepresented in ADHD + LD cases (Table 4) and their parents (*χ* ^2^ = 4.21; *P* = 0.04; power = 54% *α* at 5%). Furthermore, in ADHD + LD, “GG” genotype showed higher frequencies as compared to the control population (*χ* ^2^ = 10.1; *P* = 0.006). Lack of any association was noticed for other co-morbid conditions (Table 4).

For rs740603 (Table 4), the “G” allele (*χ* ^2^ = 3.89, *P* = 0.04) and “GG” genotype (*χ* ^2^ = 8.35, *P* = 0.015, power = 82%  *α* at 5%) were overrepresented in ADHD + ODD when compared to control. In ADHD + MD also, “G” allele (*χ* ^2^ = 7.14, *P* = 0.007) and “GG” genotype (*χ* ^2^ = 17.8, *P* < 0.0001, power = 98%  *α* at 5%) showed significant overrepresentation. Statistically significant overtransmission of the “G” allele (*P* = 0.03; power = 57%  *α* at 5%) from parents to ADHD + MD was also noticed (Table 5). For this site, both population- and family-based data showed high OR in ADHD + MD.

### 3.5. Epistatic Interaction

**Figure 1:**
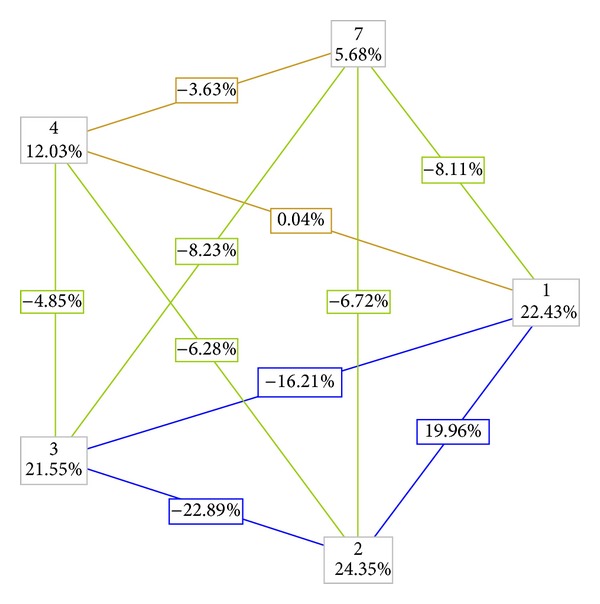
Two-way gene-gene interaction analyzed for different sites using case-control dataset. All the positive IG values in the nodes indicate independent main effect of all the markers. All the lines with negative IG values indicate redundancy or lack of any synergistic interaction between the markers. 1—rs3837091, 2—rs3735273, 3—rs1799732, 4—rs4646984, and 7—rs740603.

Gene-gene interaction analysis by MDR describes percentage of entropy (information gain—IG) by each factor or by 2-way interaction; nodes indicate independent main effect, while connecting lines between the nodes indicate interactive effect contributed by pairwise combinations. All the positive values indicate a gain in effect, whereas negative values indicate redundancy or lack of any synergistic effect. In the present study, positive nodal IG values obtained by case-control analysis indicate significant main effect of rs3735273 followed by rs3837091, rs1799732, rs4646984, and rs740603 in ADHD (Figure 1). MDR analysis of case-control data revealed strong interaction (TBA = 0.755, CVC = 10, *P* < 0.000) between rs3837091, rs1799732, rs4646984, and rs740603 (summarized in Table S5; only the best models are shown).

Gene-gene interaction analysis using family-based data (Table 6) revealed significant interaction between rs3837091 and rs1799732 only after correction for multiple testing (*P* = 0.04).

In ADHD comorbidity group, rs3837091 exhibited independent main effect followed by rs3735273, rs1799732, rs4646983, and rs740603 (Figure S1A). Though interaction between rs3837091, rs17997332, rs740603 showed a trend to be significant (*P* = 0.008), the CVC value was insignificant (Table S6). 

For ADHD + CD (Figure S1B), we have noticed significant main effect of rs3837091 followed by rs3735273, rs4646983, rs1799732, and rs4646984. No interaction was noticed between the sites for this group (Table S6). 

In ADHD + LD, rs3837091 showed significant main effects followed by rs3735273, rs1799732, rs4646983, and rs4646984 (Figure S1C). In this group also, no significant interaction between the sites was noticed (Table S6). 

ADHD + MD cases (Figure S1D) exhibited significant main effect for rs3837091 followed by rs4646983, rs740603, rs4646984, and rs1799732. Two locus interaction analyses revealed lack of significant interaction (Table S6). 

In the ADHD + ODD (Figure S1E), independent main effects were observed for rs3837091 followed by rs740603, rs165599, rs3735273, and rs1799732. Positive values for the corresponding connecting lines among DDC (rs3837091 and rs3735273), DRD2 (rs1799732), and COMT (rs165599 and rs740603) indicated interaction between the sites for this group (Figure S1E). Strong interaction between DDC, DRD2, and COMT was also documented from significant *P* values and CVC = 10 (Table S6). 

Analysis of family-based data by MDR-PDT failed to show any statistically significant result in any of these groups after corrections for multiple testing (Non-Fix *P* > 0.05, Table S7).

## 4. Discussion

In the present investigation on Indo-Caucasoid population, association of nine gene variants with ADHD and its associated co-morbid features were explored. rs3837091, rs1801028, rs4646984, rs4646983, rs740603, and rs165599 have been investigated previously in different ethnic groups for association with ADHD [12–14, 18, 22, 25–28]. Association studies have also shown contribution of rs3735273 and rs1799732 in nicotine and alcohol dependence, respectively [29, 30]. Since ADHD related behavioral attributes and conduct problems were reported to share a common genetic etiology and nicotine as well as alcohol addiction is often detected in adults with ADHD [2, 19, 20, 45], we have analyzed these sites for the first time in association with ADHD in the Indo-Caucasoid probands; independent allelic associations or transmission of different variants were noticed in subjects with ADHD, ADHD + CD, ADHD + LD, ADHD + ODD, and ADHD + MD. 

### 4.1. *DDC*

Enzyme encoded by the* DDC *gene catalyzes biosynthesis of three crucial neurotransmitters: (1) decarboxylation of L-3,4 dihydroxyphenylalanine (L-DOPA) to dopamine, (2) 5-hydroxytryptophan (5HTP) to serotonin, and (3) L-tryptophan to tryptamine. Both DA and serotonin neurotransmitter systems have been reported to be altered in ADHD [24], making DDC a good candidate gene for the disorder. Functional brain imaging studies showed increased DDC activity in the midbrains of ADHD children and decreased activity in the prefrontal regions in ADHD adults [46]. Genome-wide association scan confirmed association of *DDC *with ADHD in a number of Caucasian populations [21]. In the Chinese Han population, rs3837091 AGAG insertion/deletion in the exon 1 of *DDC* showed association with ADHD inattentive subtype [18]. In Spanish ADHD cases, *DDC* variants showed association with both childhood and adult ADHD [22], while in Irish ADHD subjects, a marginally significant overtransmission was reported [25]. rs3735273 was investigated earlier in association with nicotine dependence [29]. In the present investigation, while rs3735273 failed to show significant differences, rs3837091 “AGAG” allele showed higher transmission in ADHD probands with concomitant lower occurrence and transmission of haplotype containing the Del allele. Further analysis showed that this was due to higher maternal transmission of the “AGAG” allele specifically to male probands. Cases stratified on the basis of comorbidity revealed significant association of rs3837091 “AGAG” and rs3735273 “A” with ADHD-comorbidity and ADHD + CD, respectively. Bias in parental transmission of the “AGAG” variant was also observed, paternal in ADHD-comorbidity and maternal in ADHD + CD. In ADHD + MD, the “AGAG/AGAG” genotype showed lower frequencies in families with ADHD probands. *In silico* analysis of rs3837091 and rs3735273 by F-SNP failed to show any alteration in function of the DDC gene. Based on the biased maternal transmission, we may infer that rs3837091 may have some role in the etiology of ADHD, especially in male probands, and could be the reason for higher occurrence of ADHD + CD. It can be speculated that rs3837091 is in association with another functional site in *DDC* and further investigation is warranted to find out the actual role of *DDC* in the etiology of ADHD.

### 4.2. *DRD2*

Pharmacological intervention of several neuropsychiatric and neurologic disorders essentially relies on the modulation of function of the DRD2 receptor. SNPs in the *DRD2* gene have shown association with ADHD in probands from Finland [26]. Associations have also been reported in Brazilian [29] as well as Spanish [47] schizophrenics and Arabian addicts [48]. Since this gene may play a role in behavioral attributes, we have explored association of three functional variants, rs1800496, rs1801028, and rs1799732, with ADHD. A proline to serine substitution at codon 309 caused by C > T transition, rs1800496, was predicted to play role in protein coding, splicing regulation, and posttranslational modification (F-SNP). An earlier report also hypothesized that this substitution may cause impairment in modulating adenylate cyclase activity [49]. However, the “A” allele frequency was reported to be very low (0.002) in the Caucasian population [50]. In the exon 7 rs1801028, a C > G transition altering the 311 codon causes a serine to cysteine substitution; the Cys311 variant was reported to have decreased affinity for DA [49]. This variant was also found to alter protein coding, splicing regulation, and posttranslational modification (F-SNP). In the Caucasian population, frequency of the “G” allele was found to be 0.03 [50], and association analysis with ADHD failed to show any significance [51]. In the present investigation on Indo-Caucasoid population, both rs1800496 and rs1801028 were monomorphic for the wild type “C” allele, and thus, no association with ADHD could be ascertained. 

Another functional variant in the *DRD2,* -141C Ins/Del variant, rs1799732, alters transcriptional activity of the promoter thus regulating expression of the receptor [52] and has been reported to influence D2 receptor density in the striatum [53]. Response to antipsychotic drugs was also found to be affected by rs1799732 [54]. While no published literature on association of this variant with ADHD was observed, the -141C insertion allele showed association with alcohol dependence in Indian males [30]. Frequency of the “Del” allele was reported to be 0.14 in the Caucasian population [50], which is comparable with the frequency obtained in the present study on the Indo-Caucasoid population (0.12). Our pioneering analysis on rs1799732 in association with ADHD revealed nominal bias for the “C” allele in the probands by both population- and family-based analyses, along with statistically significant occurrence and transmission in ADHD + LD. Maternal overtransmission was also noticed in ADHD + LD group. Further, the “CC” genotype showed statistically significant higher occurrence in ADHD + LD and ADHD + MD. On the basis of the present data, it may be inferred that rs1799732 could be important for the etiology of ADHD associated LD and MD and may turn out to be useful for pharmacological as well as psychological interventions that directly hit specific neurophysiological mechanisms compromised in ADHD probands.

### 4.3. *DRD4*

DRD4 receptor is predominantly expressed in the frontal lobe regions of the brain, a region thought to be involved in the etiology of ADHD [3]. Association studies also indicate *DRD4* as a candidate gene for ADHD [5, 12–15, 21]. Extensive work has been done on the exon 3 48 bp variable number of tandem repeats, and meta-analysis of more than 30 published reports revealed that the higher repeat variant (7R), that reduces sensitivity to DA, increases risk for the disorder [15, 21]. In the Indo-Caucasoid ADHD probands, we have also observed significant association of the higher repeats [5]. Another repeat variant rs4646984, located about 1.2 kb upstream of the initiation codon and affecting transcriptional activity of the promoter [14], showed nominal association of the duplicated allele in Caucasian population from Norway, Spain [55], and USA [14, 56]. On the other hand, haplotypes containing the single repeat allele have shown higher frequency in Caucasian ADHD probands from Hungary [13]. A study on ADHD subjects from Taiwan also showed negative association with the duplicated allele [57]. A 12 bp repeat variant near the junction of the extracellular domain of the receptor, speculated to alter agonist binding and signal transduction [58], was also studied in limited number of Indo-Caucasoid ADHD (*N* = 70) and Italian delusional disorder patients (*N* = 59), respectively [12, 59]. In the present investigation, we have replicated analysis of these two repeat polymorphisms; association of the single repeat alleles of rs4646983 was noticed with ADHD. Cases with comorbidities like CD, ODD, and MD showed significantly higher frequency of the single repeat variant. rs4646984 single repeat allele also showed association with ADHD + CD (OR = 2.58). Over representation of the double repeat (2R) allele of rs4646983 (*P* = 0.04; power = 54%  *α* at 0.05) along with higher frequency of the 2R-2R haplotype in control samples (*P* = 0.05) indicates some protective role of this allele in the studied population. Whether this diversity in allelic association, in absence of any allelic flip, is due to a difference in association with the disorder or is generated due to type I error in different studied population merits further investigation in large cohort of subjects.

### 4.4. *COMT*

COMT helps in the metabolism of DA, adrenalin, and norepinephrine and has been implicated in the etiology of substance abuse, schizophrenia, and novelty seeking, as well as ADHD. A number of investigations have been carried out on a functional variant, Val/Met polymorphism, at codon 158 [19, 20], and studies in Indo-Caucasoid ADHD probands [5] as well as meta-analysis failed to support any association [60]. A G > A substitution rs740603 in the intron 1 of *COMT* gene, predicted to alter transcriptional regulation (F-SNP), though failed to show any association with ADHD in Caucasian subjects from Finland [26] and Ireland [20], a haplotype consisting of the “A” allele was reported to provide protection towards nicotine dependence in the African-American population (*P* = 0.0005) [61]. Another G > A transition rs165599 at the 3′UTR of *COMT*, predicted to affect gene expression [62], showed association with ADHD and obsessive compulsive disorder in Jews from Israel [27]. On the other hand, in British Caucasian ADHD children, rs165599 revealed no significant association [63]. The G-A haplotype consisting of rs4680-rs165599 showed higher occurrence in patients with anxiety spectrum phenotypes [64]. Sexually dimorphic effects of *COMT* haplotypes in boys and girls [65] and strong association with severity of hyperactivity symptoms [66] have also been reported. Our analysis revealed statistically significant bias in transmission of the rs740603 “G” allele to ADHD and ADHD + MD probands; the biased transmission was paternal in nature (*P* = 0.03), while maternal transmission to male probands was nominal only (*P* = 0.09). Marginally significant higher occurrence of the “G” was also observed in ADHD + ODD by population-based analysis. Higher occurrence of the “G” allele as well as “GG” genotype of rs165599 was also noticed in ADHD + LD probands. On the other hand, haplotype analysis showed a nominal bias in overtransmission of rs165599-rs740603 “G-A” (*P* = 0.04) which failed to be significant by case-control comparison. Earlier investigators reported an association of rs165599 “A” with anxiety spectrum disorder [64]. Since only a few Indian ADHD probands reported anxiety disorder further investigation, in extended number of samples, is warranted to find out whether protection to anxiety is conferred by the rs165599 “G” allele in this population. Moreover, contribution of the rs740603 “G” in ADHD also merits further exploration based on earlier report of protection to nicotine dependence [61].

### 4.5. Epistatic Interaction

In an earlier investigation on the Indo-Caucasoid ADHD probands, we have noticed additive effects of *DBH* rs1108580 and *DRD4* rs1800955, while the *DRD4* exon 3 VNTR, *DAT1* 3′UTR and intron 8 VNTR, *MAOA* u-VNTR, rs6323, *COMT* rs4680, rs362204, *DBH* rs1611115, and rs1108580 were found to exert strong independent effects [5]. Investigation on young adults from USA revealed lack of significant interaction between *DRD4* and *DAT1* (*SLC6A3*), while monoaminergic system genes showed significant interaction with ADHD symptoms [67]. On the other hand, an interaction between *DRD2-DRD4* was found to be associated with development of CD and adult antisocial behavior in males [68]. In a more recent study, no epistatic interaction was found between *COMT* and *DRD4* [69]. Alternatively, an interaction between functional variants in *DRD2* and *COMT* was found to hamper working memory [70]. In the present investigation, interactive effect of *DRD2* and *COMT* was noticed in ADHD + ODD, while in other groups, independent main effects of these sites were observed. Statistically significant interaction of *DDC* rs3837091 with *DRD2* rs1799732, *DRD4* rs4646984, and *COMT* rs740603 was also noticed by population-based analysis. Further, interaction of *DDC* rs3837091 with *DRD2* rs1799732 was strong in families with ADHD probands; the *P* value remained statistically significant even after correction for multiple testing. *DRD4* and *DDC* also exhibited significant main effects. While both DDC and COMT are important for neurotransmitter metabolism, COMT also plays vital roles in catecholestrogens and catechol-containing flavonoids. Furthermore, ADHD is hypothesized to be caused by an interaction of different genetic as well as environmental factors. It may be quite probable that the variants we found to be associated with ADHD have relatively small effect sizes keeping with the multifactorial polygenic etiology of ADHD [15, 17, 18, 21]. The other question that remains to be answered is whether the traits of ADHD are affected by haploinsufficiency for some of these alleles.

Altered dopaminergic neurotransmission is implicated in ADHD based on the presenting clinical features of probands, available animal models, and pharmacotherapeutics [3–6, 10, 46]. In the present study on Indo-Caucasoid ADHD probands, both population- and family-based analyses revealed higher transmission as well as independent effect of *DRD2* rs1799732 “C” allele. Decreased frequency of the rs1799732 “Del” allele was speculated to contribute to an elevated DRD2 density leading to DA hyperactivity [71]. In vivo experiments in mice showed that DRD2 over expression in the striatum impacts DA levels, rates of DA turnover, and activation of D1 receptors in the prefrontal cortex, the brain structure mainly associated with working memory [72]. Further, altered expression of *DRD2* and *COMT* was found to hamper working memory, a trait affected in ADHD probands [70]. On the basis of the above observations, we infer that the eastern Indian ADHD probands may have an altered DA signaling.

## 5. Conclusion

This association analysis on Indo-Caucasoid subjects with ADHD explored gene variants studied for association with different behavioral disorders. In this preliminary investigation, with limited number of ADHD probands, we have also studied association with different co-morbid conditions that are frequently observed in ADHD patients. The suggested reason for these comorbidities to be so common in ADHD subjects was hypothesized to be due to sharing of a number of gene variants [24]. As a support to the aforesaid fact, we have noticed higher frequencies and bias in transmission of *DDC, DRD2, DRD4,* and *COMT* variants in individuals with ADHD and those exhibiting different co-morbid conditions. In our earlier investigation in this ethnic group, we have observed a trend for alteration in dopaminergic neurotransmission in ADHD probands [5, 12]. The present study also indicates involvement of gene variants which may hamper catecholaminergic neurotransmission. Further investigation on functional, behavioral, and environmental attributes, incorporating larger sample sizes, is warranted to understand the complex disease etiology.

## Supplementary Material

Table S1: Details on the procedure used for genotyping the studied sites.
Table S2: Allelic transmission from parents to probands.
Table S3: Case-control analysis of haplotypic frequencies.
Table S4: Transmission of haplotypes from parents to probands.
Table S5: Gene-gene interaction analyzed using case-control dataset.
Table S6: Gene-gene interaction analyzed in ADHD cases with co-morbid features.
Table S7: Gene-gene interaction analyzed in families with ADHD probands exhibiting various co-morbid features.
Figure S1: Gene-gene interaction analyzed for co-morbid disorders using case-control dataset.

## Figures and Tables

**Table 1:** Comparative analysis of allelic frequencies in ADHD probands, their parents, and controls.

Gene	Site ID	Allele	Control (*N* = 180)	Case (*N* = 170)	*χ* ^2^ (*P*)	OR (95% CI)	Parent (*N* = 303)	*χ* ^2^ (*P*)	OR (95% CI)
DDC	rs3837091	Del	0.37	0.30	2.4 (0.1)	1.56 (0.87–2.79)	0.40	0.35 (0.6)	0.88 (0.49–1.56)
		AGAG	0.63	0.70			0.60		
	rs3735273	G	0.75	0.71	0.92 (0.33)	1.23 (0.66–2.29)	0.70	1.6 (0.21)	1.29 (0.68–2.40)
		A	0.25	0.29			0.30		

DRD2	rs1800496	C	1.00	1.00	0.0 (1.0)	—	1.00	0.0 (1.0)	—
		T	0.00	0.00			0		
	rs1801028	C	1.00	1.00	0.0 (1.0)	—	1.00	0.0 (1.0)	—
		G	0.00	0.00			0		
	rs1799732	C	0.88	0.90	0.93 (0.33)	0.81 (0.33–1.98)	0.89	0.28 (0.60)	0.91 (0.38–2.17)
		Del	0.12	0.10			0.11		

DRD4	rs4646984	1 repeat	0.25	0.32	2.83 (0.09)	0.71 (0.38–1.31)	0.33	**4.61 ** **(0.03)**	0.68 (0.37–1.25)
		2 repeat	0.75	0.68			0.67		
	rs4646983	1 repeat	0.08	0.14	**4.2 ** **(0.04)**	0.53 (0.21–1.34)	0.14	**5.18 ** **(0.02)**	0.53 (0.22–1.33)
		2 repeat	0.92	0.86			0.86		

COMT	rs165599	G	0.34	0.39	1.06 (0.30)	0.81 (0.45–1.43)	0.38	1.67 (0.19)	0.84 (0.47–1.50)
		A	0.66	0.61			0.62		
	rs740603	G A	0.48 0.52	0.54 0.46	2.11 (0.14)	0.79 (0.45–1.37)	0.51	0.64 (0.42)	0.89 (0.51–1.54)
							0.49		

NB: significant *P* values are presented in bold.

**Table 2:** Genotypic frequencies in controls compared with that of ADHD probands and their parents.

Site	Genotypes	Control (*N* = 180)	*P* value for HWE	Case (*N* = 170)	*P* value for HWE	*χ* ^2^ (*P*)	Parent (*N* = 310)	*P* value for HWE	*χ* ^2^ (*P*)
rs3837091	(Del/Del)	0.19	0.06	0.13	0.16	1.64 (0.44)	0.17	0.08	0.16 (0.93)
	(Del/AGAG)	0.36		0.35			0.36		
	(AGAG/AGAG)	0.45		0.52			0.47		

rs3735273	GG	0.57	0.41	0.49	0.84	1.42 (0.49)	0.51	0.14	1.63 (0.44)
	GA	0.35		0.43			0.38		
	AA	0.08		0.08			0.11		

rs1800496	CC	1.00	—	1.00	—	—	1.00	—	—
	CT	0.00		0.00			0.00		
	TT	0.00		0.00			0.00		

rs1801028	CC	1.00	—	1.00	—	—	1.00	—	—
	CG	0.00		0.00			0.00		
	GG	0.00		0.00			0.00		

rs1799732	CC	0.78	0.17	0.83	0.14	0.826 (0.662)	0.80	0.13	0.252 (0.89)
	C/Del	0.19		0.15			0.18		
	Del/Del	0.03		0.02			0.02		

rs4646984	1R1R	0.07	1.00	0.09	0.85	0.80 (0.67)	0.10	1.00	1.21 (0.55)
	1R2R	0.40		0.44			0.44		
	2R2R	0.53		0.47			0.46		

rs4646983	1R1R	0.00	0.60	0.04	0.32	4.80 (0.09)	0.03	0.20	3.70 (0.16)
	1R2R	0.19		0.23			0.23		
	2R2R	0.81		0.73			0.74		

rs165599	GG	0.06	0.03	0.12	0.06	1.19 (0.55)	0.14	0.78	3.59 (0.17)
	GA	0.56		0.57			0.50		
	AA	0.38		0.31			0.36		

rs740603	GG	0.26	0.18	0.30	0.86	1.69 (0.43)	0.24	0.37	1.84 (0.40)
	GA	0.44		0.48			0.53		
	AA	0.30		0.22			0.23		

**Table 3:** Analysis of allelic transmission from parents to probands (*N* = 170).

Site	Allele	Transmitted (%)	Not Transmitted (%)	*χ* ^2^ (*P* value)	Relative Risk (95% CI)
rs3837091	Del	0.35	0.65	**6.64** **(0.01)**	0.54 (0.40–0.73)
	AGAG	0.65	0.35		

rs3735273	G	0.47	0.53	0.22 (0.63)	0.89 (0.67–1.17)
	A	0.53	0.47		

rs1799732	C	0.53	0.47	0.21 (0.65)	1.28 (0.85–1.49)
	Del	0.47	0.53		

rs4646984	1 R	0.52	0.48	0.10 (0.75)	1.08 (0.82–1.43)
	2 R	0.48	0.52		

rs4646983	1 R	0.49	0.51	0.02 (0.89)	0.96 (0.73–1.27)
	2 R	0.51	0.49		

rs165599	G	0.46	0.54	0.59 (0.44)	0.85 (0.64–1.13)
	A	0.54	0.46		

rs740603	G	0.62	0.38	**5.24** **(0.02)**	1.63 (1.22–2.19)
	A	0.38	0.62		

NB: significant *P* values are presented in bold.

**Table 4:** Case-control analysis of allelic frequencies in ADHD probands with various co-morbidities.

Site	ADHD comorbidity (*N* = 42)		ADHD + CD (*N* = 33)		ADHD + LD (*N* = 42)		ADHD + ODD (*N* = 24)		ADHD + MD (*N* = 20)	
	*χ* ^2^ (*P*)	OR (95% CI)	*χ* ^2^ (*P*)	OR (95% CI)	*χ* ^2^ (*P*)	OR (95% CI)	*χ* ^2^ (*P*)	OR (95% CI)	*χ* ^2^ (*P*)	OR (95% CI)
rs3837091	4.67 (**0.03**)	1.97 (1.06–3.65)	0.00 (0.77)	1.09 (0.61–1.94)	0.20 (0.66)	1.14 (0.64–2.04)	0.00 (0.88)	0.96 (0.54–1.70)	3.44 (0.06)	0.59 (0.33–1.03)
rs3735273	0.00 (1.00)	1 (0.53–1.90)	6.49 (**0.01**)	2.17 (1.19–3.97)	0.41 (0.52)	1.23 (0.66–2.29)	0.00 (0.87)	0.95 (0.50–1.81)	3.02 (0.08)	1.71 (0.93–3.16)
rs1799732	0.02 (0.89)	1.1 (0.47–2.54)	0.59 (0.44)	1.51 (0.61–3.71)	4.64 (**0.03**)	2.84 (0.97–8.29)	0.21 (0.64)	0.85 (0.38–1.89)	1.96 (0.16)	2.84 (0.97–8.29)
rs4646984	0.12 (0.73)	1.11 (0.59–2.09)	4.39 (**0.04**)	1.91 (1.05–3.51)	1.67 (0.20)	1.47 (0.79–2.73)	1.18 (0.28)	1.41 (0.76–2.62)	0.003 (0.95)	1 (0.53–1.90)
rs4646983	0.41 (0.52)	1.42 (0.55–3.69)	4.70 (**0.03**)	2.52 (1.04–6.11)	3.16 (0.08)	2.03 (0.82–5.03)	4.75 (**0.03**)	2.52 (1.04–6.11)	4.29 (**0.04**)	2.88 (1.20–6.88)
rs165599	2.03 (0.15)	1.46 (0.83–2.60)	0.55 (0.46)	1.24 (0.70–2.20)	4.21 (**0.04**)	1.72 (0.97–3.04)	0.08 (0.78)	0.91 (0.50–1.64)	0.01 (0.91)	0.95 (0.53–1.72)
rs740603	0.41 (0.52)	1.17 (0.67–2.04)	0.09 (0.76)	1.12 (0.65–1.96)	0.03 (0.88)	1.08 (0.62–1.88)	3.89 (**0.04**)	1.76 (1.0–3.09)	**7.14** **(0.007)**	2.51 (1.41–4.47)

NB: significant *P* values are presented in bold.

**Table 5:** Analysis of allelic transmission in ADHD probands with different co-morbidities.

Site	ADHD comorbidity		ADHD + CD		ADHD + LD		ADHD + ODD		ADHD + MD	
	*χ* ^2^ (*P*)	OR (95% CI)	*χ* ^2^ (*P*)	OR (95% CI)	*χ* ^2^ (*P*)	OR (95% CI)	*χ* ^2^ (*P*)	OR (95% CI)	*χ* ^2^ (*P*)	OR (95% CI)
rs3837091	7.60 (**0.006**)	0.35 (0.25–0.50)	5.43 (**0.01**)	0.61 (0.45–0.82)	0.67 (0.41)	0.72 (0.54–0.96)	0.53 (0.47)	0.69 (0.52–0.93)	0.00 (1.00)	1.00 (0.76–1.32)
rs3735273	0.04 (0.84)	0.92 (0.06–14.83)	0.17 (0.68)	0.92 (0.70–1.22)	0.14 (0.70)	0.85 (0.64–1.12)	1.36 (0.24)	2.03 (1.49–2.77)	0.09 (0.76)	1.22 (0.92–1.62)
rs1799732	0.37 (0.55)	0.69 (0.20–2.29)	0.44 (0.51)	1.56 (0.42–5.87)	7.49 (**0.006**)	6.33 (1.35–29.68)	0.70 (0.40)	0.63 (0.21–1.90)	0.22 (0.64)	1.55 (0.24–9.85)
rs4646984	0.17 (0.68)	0.84 (0.38–1.89)	5.14 (**0.02**)	2.58 (1.12–5.93)	1.41 (0.24)	1.6 (0.73–3.5)	0.13 (0.72)	0.88 (0.43–1.78)	1.60 (0.20)	0.52 (0.19–1.44)
rs4646983	1.70 (0.19)	0.50 (0.17–1.44)	1.81 (0.17)	2.53 (0.62–10.63)	0.32 (0.57)	1.38 (0.45–4.21)	0.90 (0.34)	1.85 (0.51–6.67)	1.11 (0.29)	3.22 (0.32–32.89)
rs165599	1.64 (0.20)	1.60 (0.78–3.29)	1.64 (0.20)	0.58 (0.25–1.34)	0.03 (0.86)	1.07 (0.52–2.19)	1.27 (0.26)	0.60 (0.25–1.46)	1.59 (0.21)	0.53 (0.19–1.44)
rs740603	1.00 (0.32)	1.50 (0.68–3.27)	0.35 (0.55)	0.79 (0.36–1.72)	1.48 (0.22)	0.66 (0.33–1.30)	0.48 (0.49)	1.37 (0.56–3.39)	4.46 (**0.03**)	2.73 (1.06–7.03)

NB: significant *P* values are presented in bold.

**Table 6:** Gene-gene interaction analyzed by MDRPDT using family-based data of all ADHD cases.

Two-locus model	MDR-PDT	FixP	NonFixP
[1 3]	4.627	0.002	0.04
[1 5]	4.326	0.003	0.08
[3 5]	4.454	0.002	0.072

1—rs3837091, 2—rs3735273, 3—rs1799732, 4—rs4646984, 5—rs4646983, 6—rs165599, and 7—rs740603. No. of attributes = 7; MDR-PDT: MDR-pedigree disequilibrium test; FixP: does not control for multiple tests; Non FixP: controlling for multiple testing.
