# Supplementary material for: Catecholaminergic Gene Variants: Contribution in ADHD and Associated Comorbid Attributes in the Eastern Indian Probands
Source: Biomed Res Int. 2013 Sep 19;2013:918410. doi: 10.1155/2013/918410 (PMC3791561; doi:10.1155/2013/918410)
Supplement: Supplementary file 1 — Table S1: Details on the procedure used for genotyping the studied sites. Table S2: Allelic transmission from parents to probands. Table S3: Case-control analysis of haplotypic frequencies. Table S4: Transmission of haplotypes from parents to probands. Table S5: Gene-gene interaction analyzed using case-control dataset. Table S6: Gene-gene interaction analyzed in ADHD cases with co-morbid features. Table S7: Gene-gene interaction analyzed in families with ADHD probands exhibiting various co-morbid features. Figure S1: Gene-gene interaction analyzed for co-morbid disorders using case-control dataset. [file 918410.f1.docx]

**Table S1. Oligonucleotide sequences and amplification conditions for the sites studied.**

| Site | Oligonucleotide sequences | PCR amplification | Genotyping |
| --- | --- | --- | --- |
| rs3837091 | Sense: 5’-ggggaggcagacactctgt-3’  Antisense: 5’-tagaatcaaccggcaaacct - 3’ | 20 µl reaction mix containing 75–100 ng genomic DNA, 1X Thermopol Buffer (Genei, India), 1.5mM MgSO_4_, 200mM dNTPs, 10 pmoles of each primer and 1U Taq polymerase (Genei, India) was amplified for 35 cycles of denaturation at 94°C for 45 sec, annealing at 60°C for 45 sec and extension at 72°C for 30 sec followed by a final extension at 72°C for 5min. | 5 µl amplicon was analyzed by 20% PAGE. The deletion variant appeared as 204 bp while the wild type was 208 bp. |
| rs3735273 | Sense: 5’- ccgatcaggtgagtgtgttgt -3’  Antisense: 5’-ggatgctgtttggtttggtttgaat- 3’ | 20 µl reaction mix containing 75–100 ng genomic DNA, 1X Thermopol Buffer (Genei, India), 2 mM MgSO_4_, 200mM dNTPs, 10 pmoles of each primer and 1U Taq polymerase (Genei, India) was amplified for 35 cycles of denaturation at 94°C for 30 sec, annealing at 57°C for 40 sec and extension at 72°C for 30 sec followed by a final extension at 72°C for 5min. | 5 µl PCR amplicon was digested for 16 hrs with *BstXI* restriction enzyme in 1X NEB Buffer 3 (New England Biolabs, UK) at 37°C and products resolved in `12% PAGE. Wild type homozygous variant appeared as 95 bp while two bands of 56 bp and 39bp were obtained for the homozygous mutant type. |
| rs1800496  rs1801028 | Sense: 5’- aggagctggagatggagatg -3’  Antisense: 5’- caatcttggggtggtctttg -3’ | 20 µl reaction mix containing 75–100 ng of genomic DNA, 1X Buffer B (Genei, India), 1.0 mM MgCl_2_, 200mM dNTP mix, 10 pmoles of each primer and 1 U Taq polymerase (Genei, Bangalore, India) was amplified for 35 cycles of denaturation at 94°C for 40 sec, annealing at 59°C for 30 sec, extension at 72°C for 30 sec and a final extension at 72°C for 5min. | Amplicons were genotyped by DNA sequence analysis in ABI 3130 genetic analyzer . |
| rs1799732 | Sense 5’-tcctctccttgcctgacttg- 3’  Antisense 5’-ccaccaaaggagctgtacct- 3’ | 20 µl reaction mix containing 75–100 ng of genomic DNA, 1X Thermopol buffer (Genei, India), 1.5 mM MgSO_4_, 200mM dNTP mix, 10 pmoles of each primer and 1 U Taq polymerase (Genei, Bangalore, India) was amplified for 35 cycles of denaturation at 94°C for 30 sec, annealing at 60°C for 40 sec and extension at 72°C for 30 sec followed by a final extension at 72°C for 5min. | PCR product was digested at 60°C for 16 hrs in 10μl reaction mixture containing 1U BstNI, 1X NEB Buffer 2, 1X BSA (NEB). Digested products were resolved in 10% PAGE. Wild type variant after digestion produced 220bp and 39bp fragments where as mutant variant of 259bp remained undigested. |
| rs4646984*^a^* | Sense 5’-gttgtctgtcttttctcattgtttccattg- 3’  Antisense 5’-gaaggagcaggcaccgtgagc-3’ | 20 µl reaction mix containing 75–100 ng of genomic DNA, 1X Taq buffer B, 1.5mM MgCl_2,_ 200µM dNTPs (Genei, Bangalore, India), 2.5 pmoles of each primers (Sigma, India), 5% glycerol, 0.001% Gelatin, 1U Taq polymerase (Genei, India) was amplified for 35 cycles of denaturation at 94°C for 30 sec, annealing at 57°C for 30 sec and extension at 72°C for 1 min 30 sec followed by a final extension at 72°C for 7min | 5 µl amplicon was analyzed in 2.5% agarose gel for size determination. The single repeat variant appeared as 428bp while the duplicated allele was 548 bp. |
| rs4646983*^b^* | Sense 5'- cgccatggggaaccgcag-3’  Antisense 5’- cggctcacctcggagtaga- 3’ | Total volume of 20 µl conatining1X Taq buffer B (Genei, India), 1.2mM MgCl_2,_ 200µM dNTPs (Genei, Bangalore, India), 2.5 pmoles of each primers (Sigma, India), ~75ng template DNA, 5% glycerol, 5% Formamide, 1U Taq polymerase (Genei, India) was amplified for 35 cycles of denaturation at 94°C for 30 sec, annealing at 59°C for 30 sec and extension at 72°C for 1 min 30 sec followed by a final extension at 72°C for 5 min. | 5 µl amplicon was analyzed in 2.5% agarose gel for size determination. The single repeat variant appeared as 285bp while the duplicated type was 297 bp. |
| rs165599 | Sense 5’-gacttgggcaccaaacattc -3’  Antisense 5’-tgcttggtcagaaaggtgtg-3’ | 20 µl reaction mix containing 75–100 ng of genomic DNA, 1X Taq buffer B (Genei, India), 1.25 mM MgCl_2,_ 200µM dNTPs (Genei, Bangalore, India), 2.5 pmoles of each primers (Sigma, India), 1U Taq polymerase (Genei, India ) was exposed to 35 cycles of denaturation at 94°C for 40 sec, annealing at 60°C for 40 sec and extension at 72°C for 40 sec followed by a final extension at 72°C for 5 min. | PCR products were digested at 37°C for 16 hrs in 20μl reaction mixture containing 2U of MspI and 1X NEB Buffer 2. Fragment sizes were analyzed in 12% PAGE.  Homozygous wild type allele after digestion produced 181bp and 54bp fragments while the mutated remained undigested at 235bp. |
| rs740603 | Sense 5’-cccagaagcttcatgctctt -3’  Antisense 5’-aggtccacattccctcctct -3’ | 20 µl reaction mix containing 75–100 ng of genomic DNA, 1X Thermopol buffer (Genei, India) 1.5 mM MgSO_4,_ 200µM dNTPs (Genei, Bangalore, India), 2.5 pmoles of each primers (Sigma, India), 1U Taq polymerase (Genei, India) was amplified for 35 cycles of denaturation at 95°C for 30 sec, annealing at 60°C for 40 sec and extension at 72°C for 30 sec followed by a final extension at 72°C for 5 min. | PCR amplicon was incubated at 37°C for 16hrs in a final volume of 10μl containing 1U of HpyCH4IV and 1X NEB Buffer 1. Fragment sizes were analyzed by 12% PAGE. Two fragments of 129bp and 91bp were detected for the mutant variant while the wild type remained undigested at 220bp. |

NB: *a-*Seaman et al., Am. J Med. Genet. vol. 88, pp. 705-709, 1999; *b-* Seaman et al., J of Exp. Zool. (Mol. Dev. Evol.), vo. 288, pp. 32-38, 2000.

| Site ID | Transmission | Allele | Transmitted (%) | Not Transmitted (%) | χ^2^  (P-Value) |
| --- | --- | --- | --- | --- | --- |
| rs3837091 | Paternal | Del | 0.39 | 0.61 | 1.99  (0.16) |
|  |  | AGAG | 0.61 | 0.39 |  |
|  | Maternal | Del | 0.30 | 0.70 | 5.26  **(0.02)** |
|  |  | AGAG | 0.70 | 0.30 |  |
|  | Maternal to male proband | Del | 0.28 | 0.72 | 6.04  **(0.01)** |
|  |  | AGAG | 0.72 | 0.28 |  |
| rs3735273 | Paternal | G | 0.49 | 0.51 | 0.03  (0.87) |
|  |  | A | 0.51 | 0.49 |  |
|  | Maternal | G | 0.45 | 0.55 | 0.27  (0.60) |
|  |  | A | 0.55 | 0.45 |  |
|  | Maternal to male proband | G | 0.48 | 0.52 | 0.03  (0.86) |
|  |  | A | 0.52 | 0.48 |  |
| rs1799732 | Paternal | C | 0.44 | 0.56 | 0.25  (0.62) |
|  |  | del | 0.56 | 0.44 |  |
|  | Maternal | C | 0.61 | 0.39 | 1.10  (0.30) |
|  |  | del | 0.39 | 0.61 |  |
|  | Maternal to male proband | C | 0.62 | 0.38 | 1.20  (0.27) |
|  |  | del | 0.38 | 0.62 |  |
| rs4646984 | Paternal | 1 R | 0.60 | 0.40 | 1.90  (0.17) |
|  |  | 2 R | 0.40 | 0.60 |  |
|  | Maternal | 1 R | 0.43 | 0.57 | 0.82  (0.37) |
|  |  | 2 R | 0.57 | 0.43 |  |
|  | Maternal to male proband | 1 R | 0.40 | 0.60 | 1.61  (0.20) |
|  |  | 2 R | 0.60 | 0.40 |  |
| rs4646983 | Paternal | 1 R | 0.52 | 0.48 | 0.04  (0.84) |
|  |  | 2 R | 0.48 | 0.52 |  |
|  | Maternal | 1 R | 0.45 | 0.55 | 0.20  (0.65) |
|  |  | 2 R | 0.55 | 0.45 |  |
|  | Maternal to male proband | 1 R | 0.47 | 0.53 | 0.05  (0.81) |
|  |  | 2 R | 0.53 | 0.47 |  |
| rs165599 | Paternal | G | 0.44 | 0.56 | 0.61  (0.43) |
|  |  | A | 0.56 | 0.44 |  |
|  | Maternal | G | 0.49 | 0.51 | 0.02  (0.88) |
|  |  | A | 0.51 | 0.49 |  |
|  | Maternal to male proband | G | 0.46 | 0.54 | 0.22  (0.64) |
|  |  | A | 0.54 | 0.46 |  |
| rs740603 | Paternal | G | 0.66 | 0.34 | 4.53  **(0.03)** |
|  |  | A | 0.34 | 0.66 |  |
|  | Maternal | G | 0.60 | 0.40 | 1.53  (0.22) |
|  |  | A | 0.40 | 0.60 |  |
|  | Maternal to male proband | G | 0.64 | 0.36 | 2.82  (0.09) |
|  |  | A | 0.36 | 0.64 |  |

**Table S2. Extended Transmission Disequilibrium Test performed for allelic transmission by parents.**

**Table S3. Comparative analysis of haplotype frequencies observed in ADHD cases and controls.**

| Combination of sites | Haplotypes | Controls  (N=180) | ADHD cases (N=170) | χ^2^ (p value) |
| --- | --- | --- | --- | --- |
| rs3837091  -  rs3735273 | Del-G | 0.27 | 0.16 | 7.92  (0.004) |
|  | Del-A | 0.46 | 0.55 | 2.90  (0.09) |
|  | AGAG-G | 0.16 | 0.14 | 0.55  (0.46) |
|  | AGAG-A | 0.11 | 0.15 | 2.69  (0.10) |
| rs4646984  -  rs4646983 | 1R-1R | 0.08 | 0.11 | 1.70 (0.19) |
|  | 1R-2R | 0.01 | 0.03 | 3.21(0.07) |
|  | 2R-1R | 0.18 | 0.22 | 0.66 (0.42) |
|  | 2R-2R | 0.73 | 0.65 | 3.76 (0.05) |
| rs165599  -  rs740603 | G-G | 0.19 | 0.23 | 1.78 (1.83) |
|  | G-A | 0.30 | 0.31 | 0.28 (0.60) |
|  | A-G  A-A | 0.18  0.35 | 0.17  0.29 | 0.07 (0.79) |
|  |  |  |  | 2.10 (0.15) |

**Table S4. Transmission pattern of haplotypes in families with ADHD probands.**

| Combinations of site | Haplotypes | Transmitted (%) | Not transmitted (%) | χ^2^  (P-Value) |
| --- | --- | --- | --- | --- |
| rs3837091  -  rs3735273 | Del-G | 0.13 | 0.39 | **10.28 (0.001)** |
|  | Del-A | 0.47 | 0.31 | 2.39 (0.12) |
|  | AGAG-G | 0.14 | 0.17 | 0.11 (0.75) |
|  | AGAG-A | 0.26 | 0.13 | 3.35 (0.07) |
| rs4646984  -  rs4646983 | 1 R-1R | 0.18 | 0.10 | 1.71 (0.19) |
|  | 1R-2 R | 0.04 | 0.11 | 2.35 (0.13) |
|  | 2R-1 R | 0.32 | 0.39 | 0.61 (0.44) |
|  | 2R-2 R | 0.46 | 0.40 | 0.34 (0.56) |
| rs165599  -  rs740603 | G-G | 0.22 | 0.22 | 0.00 (1.00) |
|  | G-A | 0.38 | 0.21 | **4.35 (0.04)** |
|  | A-G | 0.16 | 0.21 | 0.56 (0.46) |
|  | A-A | 0.24 | 0.36 | 2.06 (0.15) |

**Table S5. Gene–gene interaction analyzed for ADHD cases and controls** (only the best models are presented)**.**

| Best combination in each dimension | Training BA | Testing BA | CVC | P-value | PE |
| --- | --- | --- | --- | --- | --- |
| 2 | 0.7405 | 0.7405 | 10 | 0.000-0.001 | 0.2595 |
| 1,4 | 0.8226 | 0.8100 | 8 | 0.000-0.001 | 0.1900 |
| 1,3,4,7 | 0.8976 | 0.7556 | 10 | 0.000-0.001 | 0.2444 |

1 — rs3837091, 2 —rs3735273, 3 — rs1799732, 4 — rs4646984, 5 —rs4646983, 6 — rs165599, 7 — rs740603. No. of attributes=7; Cross-validation (CV) Intervals chosen=10; BA=balanced accuracy; CVC=Cross validation contingency; PE=Prediction error. The model with the maximum testing BA, a CVC>5 out of 10 and a minimum PE for that comparison was considered as the best model.

**Table S6. Gene–gene interaction tested for different co-morbid groups using case–control dataset** (only the best models are presented)**.**

| Groups | Best combination in each dimension | Training BA | Testing BA | P-value | CVC | PE |
| --- | --- | --- | --- | --- | --- | --- |
| ADHD - comorbidity | 1 | 0.7126 | 0.6989 | 0.008-0.009 | 10 | 0.3011 |
|  | 1,2 | 0.7647 | 0.6696 | 0.044-0.045 | 9 | 0.3304 |
|  | 1,3,7 | 0.8155 | 0.6995 | 0.008-0.009 | 6 | 0.3005 |
| ADHD+CD | 1,4 | 0.6829 | 0.5615 | 0.577-0.578 | 6 | 0.4385 |
|  | 1,2,4,5 | 0.8551 | 0.6494 | 0.127-0.128 | 8 | 0.3506 |
|  | 1,2,3,4,5 | 0.8865 | 0.6114 | 0.306-0.307 | 10 | 0.3886 |
| ADHD+LD | 1 | 0.6683 | 0.6266 | 0.162-0.163 | 10 | 0.3734 |
|  | 1,2,4 | 0.7849 | 0.5866 | 0.385-0.386 | 10 | 0.4134 |
|  | 1,2,3,4 | 0.8329 | 0.5906 | 0.361-0.362 | 7 | 0.4094 |
|  | 1,2,3,4,5 | 0.8663 | 0.6210 | 0.181-0.182 | 10 | 0.3790 |
| ADHD+MD | 1 | 0.7015 | 0.6626 | 0.174-0.175 | 10 | 0.3374 |
|  | 1,4,7 | 0.8581 | 0.6790 | 0.116-0.117 | 10 | 0.3210 |
|  | 1,3,4,7 | 0.9081 | 0.6626 | 0.174-0.175 | 10 | 0.3374 |
|  | 1,3,4,5,7 | 0.9302 | 0.6361 | 0.283-0.284 | 10 | 0.3639 |
| ADHD+ODD | 1,7 | 0.7606 | 0.6223 | 0.219-0.220 | 10 | 0.3777 |
|  | 1,3,7 | 0.8374 | 0.7470 | 0.004-0.005 | 10 | 0.2530 |
|  | 1,3,6,7 | 0.8847 | 0.6258 | 0.204-0.205 | 9 | 0.3742 |
|  | 1,2,3,6,7 | 0.9223 | 0.5758 | 0.436-0.437 | 10 | 0.4242 |

1 —rs3837091, 2 — rs3735273, 3 —rs1799732, 4 — rs4646984, 5 — rs4646983, 6 — rs165599, 7 — rs740603. No. of attributes=7; Cross-validation (CV) Intervals chosen=10; BA=balanced accuracy; CVC=Cross validation contingency; PE=Prediction error. The model with the maximum testing BA, a CVC>5 out of 10 and a minimum PE for that comparison was considered as the best model.

**Table S7. Gene–gene interaction analyzed for different co-morbid groups using family-based data.**

| Two locus model | MDR-PDT | FixP | NonFixP |
| --- | --- | --- | --- |
| No comorbidity  [1 2]  [1 3]  [1 5]  [1 6]  [1 7] | 4.263  4.36  3.748  5.095  4.341 | **0.026**  **0.002**  **0.047**  **0.003**  **0.017** | 0.225  0.203  0.433  0.095  0.208 |
| ADHD+CD  [2 4]  [3 4]  [4 6] | 4.608  3.244  4.446 | **0.016**  **0.055**  **0.042** | 0.222  0.775  0.276 |
| ADHD+LD  [1 4]  [2 3]  [2 4]  [3 4]  [3 5]  [4 5] | 4.345  3.817  4.36  3.817  3.481  3.889 | **0.019**  **0.024**  **0.022**  **0.042**  **0.038**  **0.019** | 0.178  0.355  0.176  0.355  0.517  0.328 |
| ADHD+ODD  [1 3]  [1 6]  [3 6]  [5 6] | 3.899  5.27  4.206  3.903 | **0.032**  **0.03**  **0.048**  **0.054** | 0.652  0.208  0.517  0.651 |
| ADHD+MD  [6 7] | 4.32 | **0.071** | 0.542 |

1 —rs3837091, 2 — rs3735273, 3 — rs1799732, 4 —rs4646984, 5 —rs4646983, 6 — rs165599, 7 — rs740603 No. of attributes=7; MDR-PDT: MDR-Pedigree Disequilibrium Test; FixP: does not control for multiple tests; NonFixP: controlling for multiple testing.

**
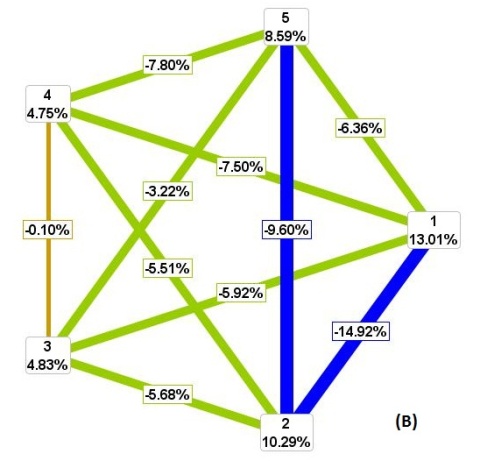

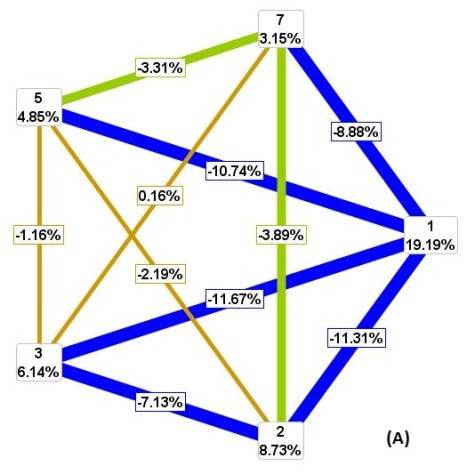
Figure S1. Gene-gene interaction analyzed for co-morbid disorders using case-control dataset.**


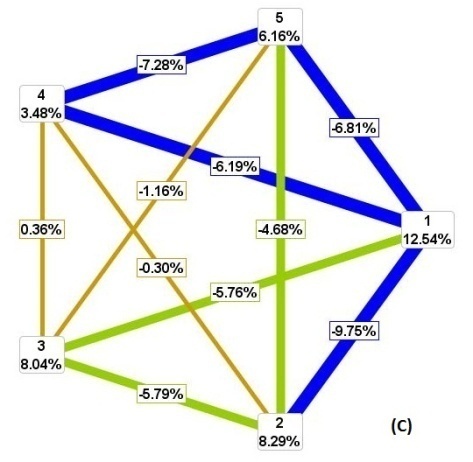

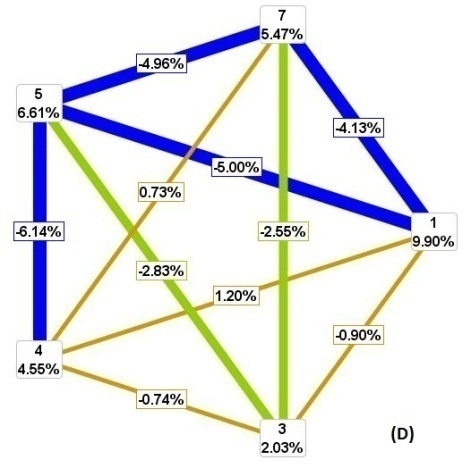


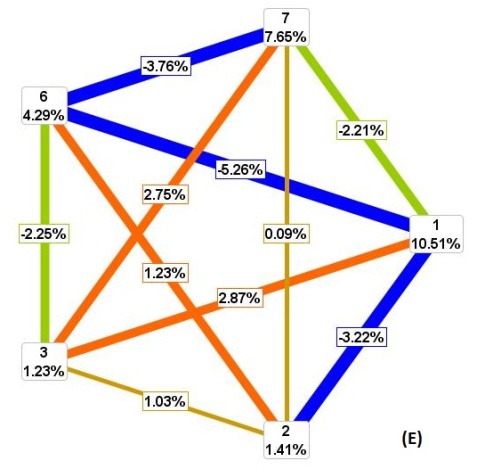


Gene-gene interaction study of (A) ADHD-comorbidity, (B) ADHD+CD, (C) ADHD+LD (D) ADHD+MD and (E) ADHD+ODD. All the positive IG values in the nodes indicate independent main effect of all the markers. All the lines with negative IG values indicate redundancy or lack of any synergistic interaction between the markers.

**Nodal No. 1­**- rs3837091, **2-** rs3735273, **3-** rs1799732, **4-** rs4646984, **5-** rs4646983, **6-** rs165599, **7-** rs740603.
